# Improving patient access to medication information: a quality improvement project on patient-centred prescribing

**DOI:** 10.1192/bjo.2021.600

**Published:** 2021-06-18

**Authors:** Anna Todd, Rosy Blunstone

**Affiliations:** Bethlem Royal Hospital, South London and Maudsley NHS Foundation Trust

## Abstract

**Aims:**

Ward rounds are sometimes the only opportunity for patients to discuss medication. Patient and professional feedback on an acute male inpatient ward in South London highlighted a demand for more medication information outside the formal ward round setting. We aimed to have 100% of patients meet our criteria for “Patient-Centred Prescribing” on the ward by March 2021. To fulfil criteria, all patients are offered: (1) ward round discussion, (2) written patient information leaflets (PILs), (3) informal discussion groups, all regarding medication.

The principles of this quality improvement project (QIP) were drawn from definitions of patient-centred care and standards of good practice; patients should have access to a variety of information formats, relevant to the individual, and the knowledge gained empowers patients.

Patient experience data revealed that 30% of clients answered passively to the question, “Do you feel involved in your care?” We hypothesized that medication discussion groups positively impact patients’ wellbeing, by providing a safe space that facilitates conversation surrounding medication issues.

**Method:**

We conducted weekly audits on patients whose admission duration was >7 days, and recorded fulfilment of the above criteria. At week 1, we introduced a program of weekly medication discussion groups led by members of the wider multi-disciplinary team covering a broad topic range. At week 6, we developed a rolling rota of the discussion groups and posters were displayed in advance. At week 14, all patients were offered PILs through a 1:1 interaction and this continued as routine practice. Medication discussion group feedback was obtained via questionnaires and “The Blob Tree”, a psycho-emotional assessment tool commonly used in healthcare settings.

**Result:**

In 19 weeks, the median percentage of patients who fulfilled our criteria for Patient-Centred Prescribing was 92.86%. After 11 medication discussion groups, 79.3% of questionnaire responders wanted further sessions. 88% of “The Blob Tree” responses collected inferred a positive emotional response after the group discussions and half of those noticed an improvement in their emotional state.

**Conclusion:**

This QIP was overall a success; it fulfilled a requirement to meet good standards in information sharing and became embedded in the fabric of the ward, continuing to run as part of the activities program. It demonstrated the impact of education on patients’ mental wellbeing through empowerment and peer support. As a by-product it established multidisciplinary connections and improved therapeutic relationships. Challenges included patient engagement secondary to acute mental illness or negative symptoms and maintaining project momentum following a COVID-19 outbreak.

